# Multi-gene phylogeny and morphological evidence reveal two novel species and two new records of *Acrogenospora* (*Minutisphaerales*, *Acrogenosporaceae*) from aquatic habitats in mainland China

**DOI:** 10.3897/mycokeys.130.182237

**Published:** 2026-03-26

**Authors:** Mei-Xia Wang, Jia-Xin Nie, Ye-Tao Wang, Min Xiong, Jing Wang, Hai-Yan Song, Dian-Ming Hu, Zhi-Jun Zhai

**Affiliations:** 1 Jiangxi Key Laboratory for Excavation and Utilization of Agricultural Microorganisms, Jiangxi Agricultural University, Nanchang, 1101 Zhimin Road, Nanchang, 330045, China Jiangxi Key Laboratory for Excavation and Utilization of Agricultural Microorganisms, Jiangxi Agricultural University Nanchang China; 2 Laboratory of Subtropical Biodiversity, Jiangxi Agricultural University, Nanchang, Jiangxi 330045, China Laboratory of Subtropical Biodiversity, Jiangxi Agricultural University Nanchang China; 3 College of Bioscience and Bioengineering, Jiangxi Agricultural University, Nanchang, 1101 Zhimin Road, Nanchang, 330045, China College of Bioscience and Bioengineering, Jiangxi Agricultural University Nanchang China; 4 School of Life Science, Nanchang Normal University, 330032, Nanchang, Jiangxi, China Nanchang Normal University Nanchang China

**Keywords:** *
Acrogenospora
*, *
Acrogenosporaceae
*, freshwater fungi, phylogenetic analyses, taxonomy

## Abstract

Freshwater fungi are characterized by their high species diversity and perform vital ecological roles. During our survey of Chinese aquatic microfungal diversity, four fungal strains were isolated from freshwater environments in Jiangxi and Guizhou Provinces, China. Through comprehensive morphological assessment and multi-locus phylogenetic analyses based on combined ITS, LSU, SSU, *RPB2*, and *TEF1-α* sequences, two new species, *Acrogenospora bambusae* and *A. lignicola*, were introduced. In addition, two previously described species, *A. taiwanica* and *A. thailandica*, originally described from China (Taiwan), and Thailand, respectively, are newly reported from mainland China. This study provides detailed morphological descriptions, photographic illustrations, and phylogenetic placements for each taxonomic novelty.

## Introduction

Freshwater fungi play a crucial role in nutrient and carbon cycling, the maintenance of biodiversity, and overall ecosystem functioning (Swe et al. 2009; Krauss et al. 2011). Extensive research has been conducted on freshwater fungi, with particular emphasis on their diversity, taxonomy, and phylogeny (Yang et al. 2018; Bao et al. 2020; Zhang et al. 2022; Wang et al. 2024; Xu et al. 2025). A significant contribution in this field was the establishment of the order *Minutisphaerales* by Raja et al. (2015). This order was later expanded by Jayasiri et al. (2018) to accommodate the family *Acrogenosporaceae*, based on phylogenetic evidence. The family *Acrogenosporaceae* includes one genus, *Acrogenospora*, which was introduced by Ellis (1971), with two species: *A. sphaerocephala* (the type species) and *A. carmichaeliana* (originally described as *Farlowiella carmichaeliana* by its asexual form). Later, Ellis (1972) added *A. setiformis*. Goh et al. (1998) revised the genus by transferring two species from *Farlowiella* to *Acrogenospora* based on the characteristics of solitary, aseptate conidia that are produced acrogenously from percurrently proliferating conidiogenous cells at the tips of dematiaceous, unbranched conidiophores, thereby expanding the genus to include eight species. Rossman et al. (2015) affirmed the synonymy of *Acrogenospora* and *Farlowiella*, advocating for the retention of *Acrogenospora* due to its wider usage and nomenclatural stability. Subsequent morphological and molecular studies conducted by Jayasiri et al. (2018) supported the recognition of *Acrogenospora* as the asexual morph of *Farlowiella* and established the family *Acrogenosporaceae* to accommodate the genus *Acrogenospora*. This genus was significantly expanded by Bao et al. (2020), who described seven new species from freshwater environments based on both morphology and molecular phylogeny, raising the total number of known species to twenty. Recently, nine additional species from both freshwater and terrestrial habitats, such as *A. alangii*, *A. alticampestriicola*, *A. guangxiensis*, *A. guizhouensis*, *A. hydei*, *A. keviniana*, *A. stellata*, *A. taiwanica*, and *A. terricola*, were described, exhibiting asexual morphs (Harrington et al. 2022; Tan et al. 2022; Hyde et al. 2023; Li et al. 2024; Cao et al. 2025; Kirschner 2025; Xiao et al. 2025; Xu et al. 2025).

To date, 29 species of *Acrogenospora* have been cataloged in Index Fungorum (2026) (http://indexfungorum.org/Names/Names.asp; accessed on 28 Feb. 2026). Most of them are saprophytic on decaying submerged woody substrates, and the genus exhibits a worldwide distribution, including Asia, America, Europe, and Oceania (Ellis 1972; Hughes 1978; Goh et al. 1998; Hyde et al. 2019; Bao et al. 2020; Harrington et al. 2022; Tan et al. 2022; Xiao et al. 2025; Xu et al. 2025; Table 2). The sexual morph of the genus is characterized by the formation of ascomata, which are often solitary or aggregated, erect, and superficial. These structures possess papillate or poroid ostioles, sometimes with depressed fissures, and contain eight-spored, unitunicate asci. The ascospores are hyaline to pale brown, 1–2-celled, and may be cylindrical to clavate in shape (Sivanesan 1984; Boehm et al. 2009; Tan et al. 2022; Li et al. 2024; Xu et al. 2025). In contrast, the hyphomycetous asexual morph produces large, macronematous, mononematous conidiophores that are brown and sometimes geniculate. The conidiogenous cells are terminal or intercalary, monoblastic, and produce solitary conidia that are globose, ellipsoidal, or obovoid, ranging in color from olive to dark brown (Hughes et al. 1978; Goh et al. 1998; Harrington et al. 2022; Tan et al. 2022; Hyde et al. 2023; Li et al. 2024; Cao et al. 2025; Kirschner 2025; Xiao et al. 2025; Xu et al. 2025). Although members of this genus share broad morphological similarities, they can be distinguished based on characteristics such as the pigmentation of the conidiophores, as well as the shape, size, color, presence of inner sheaths, and structure of the basal cells of the ascospores or conidia (Hughes et al. 1978; Bao et al. 2020; Tan et al. 2022).

**Table 1.** Species that used sequences in this study.

Species	Voucher/Culture	GenBank accession number				
		ITS	LSU	SSU	*RPB2*	*TEF1-α*
***Acrogenospora alangii***	**KUNCC 23–14553**	**OR557426**	**OR553807**	**OR553806**	**OR575924**	**OR575926**
	UESTCC 23.0140	OR578817	OR574254	OR574239	OR575925	OR575927
***A. alticampestriicola***	**KUCC 10428**	**OP626351**	**PQ455263**	**PQ218226**	**–**	**–**
***A. aquatica***	**MFLUCC 20–0097**	**–**	**–**	**MT340743**	**MT367159**	**MT367151**
	MFLUCC 16–0949	–	MT340732	–	MT367160	MT367152
A. bambusae*	**JAUCC 5526**	**PQ625471**	**PQ628330**	**PQ625475**	**PX570198**	**PX570205**
	JAUCC 5536	PX570397	PX570622	PX456102	PX570196	PX570203
***A. basalicellularispora***	**MFLUCC 16–0992**	**–**	**MT340729**	**–**	**–**	**–**
*A. carmichaeliana*	CBS 206.36	–	MH867287	AY541482	–	–
***A. guangxiensis***	**ZHKUCC 24–0794**	–	**PP693395**	**PP693396**	**PP695182**	**PP695182**
***A. guizhouensis***	**GZCC 22-2022**	–	**OP748933**		**OP750333**	**OP750332**
***A. guttulatispora***	**MFLUCC 17–1674**	**–**	**MT340730**	**–**	**MT367157**	–
***A. hydei***	GZCC 24**–**0022	**PV820348**	**PV856165**	**PV851429**	–	–
***A. keviniana***	GZCC 24**–**0108	**PV820349**	**PV856166**	–	–	–
A. lignicola*	**JAUCC 5532**	**PQ625470**	**PX570620**	**PQ625474**	**PX570193**	**PX570200**
	JAUCC 5534	PX570396	PX570621	PX456101	PX570194	PX570201
***A. obovoidispora***	**MFLUCC 18–1622**	**–**	**MT340736**	**MT340747**	**MT367163**	**MT367155**
***A. olivaceospora***	**MFLUCC 20–0096**	**–**	**MT340731**	**MT340742**	**MT367158**	**MT367150**
*A. sphaerocephala*	MFLUCC 16–0179	MH606233	MH606222	–	MH626448	–
***A. submersa***	**MFLUCC 18–1324**	**–**	**MT340735**	**MT340746**	**MT367162**	**MT367154**
*A. subprolata*	MFLUCC 18–1314	–	MT340739	MT340750	–	–
***A. stellata***	**AMI-SPL 1243**	**OP439740**	**OP439739**	**–**	**–**	**–**
***A. taiwanica***	**TNM 5903**	**PQ323568**	**–**	**–**	**LC842529**	**–**
	BCRC FU3153	PQ323567	PQ323569	–	LC842528	–
A. taiwanica*	JAUCC 5524	PX570394	PQ625473	PX456103	PX570197	PX570204
	JAUCC 5528	PX570395	PX570623	PX456104	PX570199	PX570206
***A. terricola***	PS3565	ON176299	ON176305	ON176286	**–**	**–**
	**PS3610**	**ON176304**	**ON176306**	**ON176287**	–	–
***A. thailandica***	**MFLUCC 17–2396**	**MH606234**	**MH606223**	**MH606221**	**MH626449**	**–**
A. thailandica*	JAUCC 5530	PQ625472	PQ625839	PX456100	PX570195	PX570202
*A. verrucispora*	MFLUCC 18–1617	–	MT340738	MT340749	MT367164	MT367156
***A. yunnanensis***	**MFLUCC 18–1611**	**–**	**MT340733**	**MT340744**	**–**	**–**
	MFLUCC 20–0099	–	MT340734	MT340745	MT367161	MT367153
***Minutisphaera aspera***	**DSM 29478**	**NR_154621**	**NG_060319**	**NG_065059**	**–**	**–**
***M. japonica***	**HHUF 30098**	**NR_119419**	**NG_042338**	**NG_064840**	**–**	**–**

Note. Ex-type strains or type materials are marked in bold. Newly generated sequences are indicated with “*”. “–”, the sequence data is unavailable. **Abbreviation: AMI-SPL**: Collection of A. Mateos and S. De la Peña, Azores, Terceira, Portugal; **BCC**: BIOTEC Culture Collection, Thailand; **BCRC**: Bioresource Collection & Research Center in Hsinchu City, Taiwan, China; **CBS**: CBS-KNAW Fungal Biodiversity Centre, Utrecht, The Netherlands; **DSM**: Leibniz Institute DSMZ-German Collection of Microorganisms and Cell Cultures GmbH, Braunschweig, Science Campus Braunschweig-Süd, Germany; **FMR**: Facultat de Medicina i Ciències de la Salut, Reus, Spain; **JAUCC**: the Culture Collection of Jiangxi Agricultural University; **JCM**: Japan Collection of Microorganism, RIKEN BioResource Center, Japan; **HHUF**: Herbarium of Hirosaki University, Japan; **KUNCC**: Kunming Institute of Botany, Chinese Academy of Sciences Culture Collection, Kunming, Yunnan, China; **MFLU**: the herbarium of Mae Fah Luang University, Chiang Rai, Thailand; **MFLUCC**: Mae Fah Luang University Culture Collection, Chiang Rai, Thailand; **PS**: the R. L. Gilbertson Mycological Herbarium at the University of Arizona (MYCO-ARIZ); **UESTCC**: University of Electronic Science and Technology Culture Collection, Chengdu, China; **TNM**: the National Museum of Natural Science, Taichung, Taiwan, China; ITS: internal Transcribed Spacer region of ribosomal RNA gene; LSU: large subunit ribosomal RNA gene; SSU: small subunit ribosomal RNA gene; *RPB2*: RNA polymerase II largest subunit 2; *TEF1-α*: translation elongation factor 1-alpha gene.

**Table 2.** Synopsis of morphological characteristics, habitats, hosts, and district compared across *Acrogenospora* species.

Species	Conidiophores		Conidia			Sequence data	Habitat /Locality	References
	Color	Size (μm)	Color	Size (μm)	Shape			
*Acrogenospora alangii*	Brown to dark brown	179–687 × 2.7–5.5	Hyaline and pale gray when young, pale to dark brown when mature	15–22 × 15–23	Spherical or subspherical	Present	Freshwater/Guizhou, China	Li et al. 2024
*A. alticampestriicola*	Brown, or dark brown	318–589 × 5–9	Olive when young, black when mature	21–30 × 20–30	Subglobose	Present	Freshwater/Yunnan and Xizang, China	Xu et al. 2025
*A. altissima*	Blackish brown to black	Up to 800 × 12–20	Dark to blackish brown	40–60 × 30–36	Broadly ellipsoidal	Absent	Freshwater/Westland, New Zealand	Goh et al. 1998
*A. aquatica*	Brown to dark brown, paler toward apex	202–250 × 7.5–9.5	Dark brown to black	29–34.5 × 24.5–31	Subprolate to broadly Ellipsoidal, with a basal cell and guttules	Present	Freshwater/Yunnan, China	Bao et al. 2020
***A. bambusae***	**Light brown to black**	**254–533 × 5.5–13**	**Hyaline when young, orange-brown at maturity**	**33–52 × 24–41**	**Mostly oval to broadly ellipsoidal**	**Present**	**Freshwater/Jiangxi and Guizhou, China**	**This study**
*A. basalicellularispora*	Brown to dark brown, paler toward apex	259–395 × 8–12	Pale orange-brown to olivaceous brown	27.5–33.5 × 21.5–25.5	Broadly obovoid to spherical, with basal cell	Present	Freshwater/Yunnan, China	Bao et al. 2020
*A. carmichaeliana*	Brown to dark brown	Up to 400 × 9–12	Brown to dark brown	19–32 × 16–23.5	Broadly ellipsoidal to obovoid	Present	Freshwater/Surrey, United Kingdom	Goh et al. 1998
*A. ellipsoidea*	Pale orange brown to mid brown	87.5–162.5 × 6.5–7.5	Dark brown	32–41 × 17–24	Ellipsoidal	Absent	Freshwater/Yunnan, China	Hu et al. 2010
*A. gigantospora*	Dark blackish brown	Up to 700 × 9–14.5	Dark brown to black (opaque)	25–55 × 21–49.5	Broadly obovoid to spherical	Absent	Freshwater/Westland, New Zealand	Hughes 1978
*A. guangxiensis*	Dark brown	280–475 × 6–10	Initially olive-green, later brown to dark brown	23–29	Spherical	Present	Freshwater/Guangxi, China	Cao et al. 2025
*A. guizhouensis*	Brown to dark brown	145–320 × 7.5–14	Brown	19–27.5 × 19.5–28.5	Subspherical	Present	Terrestrial/Guizhou, China	Hyde et al. 2023
*A. guttulatispora*	Dark brown, pale rtoward apex	294–331 × 7.5–8.6	Hyaline when young, dark brown at mature	30–33.5 26.5–28	Spherical or subspherical, with a large guttule	Present	Freshwater/Yunnan, China	Bao et al. 2020
*A. hainanensis*	Brown to dark brown, paler toward the apex	60–80 × 2–3.5	Brown	7.5–9.5 × 7–8.5	Spherical or subspherical	Absent	Terrestrial/Hainan, China	Ma et al. 2012
*A. hydei*	Dark brown at base, paler towards apex	137–356 × 8–15	Dark brown when mature	22–44 × 23–32	Subglobose, ellipsoidal to pyriform	Present	Freshwater/Guizhou, China	Xiao et al. 2025
*A. keviniana*	Dark brown at base, paler towards apex	404–588 × 8–15	Dark brown when mature	22–37 diam	Globose	Present	Freshwater /Guizhou, China	Xiao et al. 2025
***A. lignicola***	**Dark brown to black**	**149.5–352 × 4.5–7.5**	**Dark brown to black**	**26–45 × 22–36**	**Mostly broadly obovoid**	**Present**	**Freshwater/Jiangxi, China**	**This study**
*A. megalospora*	Black opaque, yellow-brown at apex	Up to 400 × 9–12	Mid to dark brown	19–32 × 13–23	Obovoid	Absent	Terrestrial/Surrey, United Kingdom	Goh et al. 1998
*A. novae-zelandiae*	Black opaque, paler at apex	Up to720 × 10–16	Mid to dark brown	26–54 × 21.5–30.5	Broadly ellipsoidal to oblong	Absent	Terrestrial/Canterbury, New Zealand	Hughes 1978
*A. obovoidispora*	Brown to dark brown, paler toward apex	209–277 × 7.5–10	Olivaceous brown to black	32.4–37.6 × 27–32	Oval to broadly obovoid	Present	Freshwater/Yunnan, China	Bao et al. 2020
*A. olivaceospora*	Dark brown to olive, paler toward apex	102–172 × 5.8–9	Olive to black	32–36.9 × 28–32.8	Subprolate to broadly ellipsoidal	Present	Freshwater/Yunnan, China	Bao et al. 2020
*A. ovalia*	Pale to mid brown	Up to 240 × 4–4.5	Mid orange-brown	24–33 × 18–22	Oval to oblong or broadly obovoid	Absent	Freshwater/Hong Kong, China	Goh et al. 1998
*A. setiformis*	Dark blackish brown	Up to 350 × 4–7	Dark reddish brown	14.5–24 × 10.5–19	Broadly ellipsoidal	Absent	Terrestrial/Ontario, Canada	Ellis 1972; Goh et al. 1998
*A. sphaerocephala*	Mid to dark brown, pale brown at apex	100–730 × 7.2–10.5	Pale to mid brown	17–30 × 15.5–30	Subspherica	Present	Freshwater/Queensland, Australia	Hughes 1978; Goh et al. 1998
*A. stellata*	Blackish brown or black	200–570 × 6–10	Dark brown, olive or blackish brown	25–30 × 15–20	Ellipsoidal or broadly ellipsoidal	Present	Terrestrial/Azores, Portugal	Tan et al. 2022
*A. submersa*	Brown to dark brown, paler toward apex	163–223 × 6.7–10	Hyaline when young, pale orange-brown to olivaceous brown at mature	28–32.5 × 25–28	Spherical or subspherical	Present	Freshwater/Yunnan, China	Bao et al. 2020
*A. subprolata*	Pale to mid brown	150–300 × 9–12	Pale orange-brown to olivaceous brown	39–46 × 30–39	Broadly ellipsoidal to subprolate	Present	Freshwater/Hong Kong, China	Goh et al. 1998
*A. taiwanica*	Dark brown	(180–) 390–680(–850) × 6–8	Dark brown	(19–)21.5–25 diam	Globose	present	Terrestrial /Taiwan, China	Kirschner 2025
***A. taiwanica***	**Dark brown**	**92.5–417 × 5–7.5**	**Hyaline when young, dark brown when mature**	**21–32.5 diam**	**Globose or subglobose**	**Present**	**Freshwater/Jiangxi, China**	**This study**
*A. terricola*	Dark brown at the base but translucent brown towards the apex	Up to 950 long	Deep brown to black	19–25 diam	Spherical to ovoid with a truncate base	present	Terrestrial/Barro Colorado Island, Panama	Harrington et al. 2022
*A. thailandica*	Pale to dark brown, paler toward the apex	850–950 × 3.5–8	Olive-green to dark brown	15.5–24.5	Spherical or subspherical	Present	Freshwater/Trat, Thailand	Hyde et al. 2019
***A. thailandica***	**Pale to dark brown**	**204.5–506 × 5–9.5**	**Olive-green to dark brown**	**21–34.5 diam**	**Spherical or subspherical**	**Present**	**Freshwater/Jiangxi, China**	**This study**
*A. verrucispora*	Brown to dark brown, paler toward apex	100–230 × 5–6	Mid to dark brown	19–21.5 diam	Spherical or subspherical	Present	Freshwater/Yunnan, China	Zhu et al. 2005; Bao et al. 2020
*A. yunnanensis*	Brown to dark brown, paler toward apex	260–391 × 8.6–12	Hyaline when young, dark brown to black at mature	23–32.5 × 22–30	Spherical or subspherical	Present	Freshwater/Yunnan, China	Bao et al. 2020

In this study, four fungal strains were isolated from decaying submerged wood collected from freshwater habitats in mainland China. Phylogenetic analyses based on Maximum Likelihood (ML) and Bayesian Inference (BI), complemented by detailed morphological observations, confirm the introduction of two novel species and report two new geographic records within the genus *Acrogenospora*. A comparative synoptic table summarizing key diagnostic characteristics of known *Acrogenospora* species is provided. These results contribute to a deeper understanding of fungal diversity and distribution in freshwater ecosystems.

## Materials and methods

### Sample collection, specimen examination, and isolation

The decaying wood samples were collected from freshwater streams in Jiangxi and Guizhou Provinces, China, between 2020 and 2022. The samples were transported to the laboratory in sealed plastic bags for further processing. Initial examination of fungal structures *in situ* was conducted using a Nikon SMZ-1270 stereomicroscope (Nikon Corporation, Japan). The fungal structures were carefully excised using a sterile syringe needle and mounted in distilled water on glass slides for microscopic observation. Morphological observations were conducted using a Nikon ECLIPSE Ni-U compound microscope (Nikon Corporation, Japan), and images were captured with a Nikon DS-Fi3 digital camera (Nikon Corporation, Japan). All microscopic measurements were taken using PhotoRuler v. 1.1 software (The Genus Inocybe, Hyogo, Japan), with scale bars included for reference. Illustrations were prepared using Adobe Photoshop CC 2017 18.1.3 software (Adobe Systems, USA). Pure cultures were obtained through single-spore isolation following the protocol described by Chomnunti et al. (2014). Germinated spores were transferred to potato dextrose agar (PDA, Beijing Bridge Technology Co., Ltd., China) supplemented with antibiotics (penicillin 100 μg/mL and streptomycin 50 μg/mL) to minimize bacterial interference and then incubated at 25 °C for 2–3 weeks. Fungal cultures have been deposited in the Culture Collection of Jiangxi Agricultural University (**JAUCC**), and the corresponding wood specimens were preserved in the Fungal Herbarium of Jiangxi Agricultural University (**HFJAU**).

### DNA extraction, PCR amplification, and sequencing

Fresh mycelia of each strain were scraped from colonies grown on PDA plates and transferred to a 2.0 mL microcentrifuge tube using a sterilized scalpel. Fungal genomic DNA was extracted using the TIANGEN Plant Genomic DNA Extraction Kit (Beijing Co. Ltd., People’s Republic of China), following the protocols in the manufacturer’s instructions.

Polymerase chain reaction (PCR) amplification was performed for the ITS, LSU, SSU, *RPB2*, and *TEF1-α* gene regions using the following primer pairs: ITS1/ITS4 for ITS (White et al. 1990), LR0R/LR7 for LSU (Hopple and Vilgalys 1999), NS1/NS4 for SSU (White et al. 1990), fRPB2-5F/fRPB2-7cR for *RPB2* (Liu et al. 1999), and EF1-983F/EF1-2218R for *TEF1-α* (Rehner and Buckley 2005). Each 25 μL reaction mixture contained 12.5 μL of 2 × Taq PCR Master Mix (Qingke, Changsha, China), 1 μL of DNA template, 1 μL of each forward and reverse primer (10 μM), and 9.5 μL of ddH_2_O. The PCR conditions followed LR0R, those described by Zhai et al. (2022). Amplified products were purified and sequenced by Qingke Biotechnology Co., Ltd. (Changsha, China) using the corresponding primers. All sequences were assembled and edited using SeqMan v. 7.1.0 (DNASTAR, Inc., Madison, WI, USA) and have been deposited in NCBI GenBank (accession numbers provided in Table 1).

### Sequence data analyses

Based on comparisons of ITS, LSU, SSU, *RPB2*, and *TEF1-α* sequences with the GenBank database, highly similar sequences were identified using BLAST (http://www.ncbi.nlm.nih.gov/blast/) searches. A total of 28 reference sequences were retrieved from GenBank based on BLAST results and recent publications (Tan et al. 2022; Hyde et al. 2023; Li et al. 2024; Cao et al. 2025; Xiao et al. 2025; Xu et al. 2025). Each gene region (ITS, LSU, SSU, *RPB2*, and *TEF1-α*) was individually aligned using the MAFFT v. 7 online service (http://mafft.cbrc.jp/alignment/server/index.html; accessed on 24 February 2026) with the iterative refinement method (Madeira et al. 2019). All alignments were visually inspected and manually refined using BioEdit v. 7.2.5 (http://www.mbio.ncsu.edu/BioEdit/bioedit.html). This process involved correcting misalignments, adjusting gaps, and trimming inaccurate sequences from both ends to ensure data quality (Hall 1999; Liu et al. 2017). The resulting alignments were then concatenated using PhyloSuite v. 1.2.2 (https://github.com/dongzhang0725/PhyloSuite; Zhang et al. 2020).

Phylogenetic analyses were conducted using both maximum likelihood (ML) and Bayesian inference (BI) approaches. ML analysis was performed with RAxML v. 7.2.6 (Stamatakis and Alachiotis 2010) under the GTR+GAMMA substitution model, with branch support evaluated by 1000 rapid bootstrap replicates. BI analysis was implemented in MrBayes v. 3.2 (Ronquist et al. 2012) using partitioned models. The best-fit nucleotide substitution models for each partition (ITS, LSU, SSU, *RPB2*, and *TEF1-α*) were selected based on the Akaike Information Criterion (AIC) in jModelTest v. 2.1.1 (Darriba et al. 2012) via the CIPRES Science Gateway (Miller et al. 2010). The selected models were as follows: TIM2ef+I+G for ITS (-lnL = 3744.6247), GTR+I+G for LSU (-lnL = 4733.9456), TPM3uf+I for SSU (-lnL = 1670.9488), TIM1+G for *RPB2* (-lnL = 4564.1123), and TIM3+G for *TEF1-α* (-lnL = 3220.1580). The Bayesian analysis was run for 10 million generations with four chains, sampling trees every 1000 generations. The first 10% of sampled trees were discarded as burn-in. Resulting trees were visualized and annotated using FigTree v. 1.4.4 (http://tree.bio.ed.ac.uk/software/figtree/; Rambaut 2018) and further edited in Adobe Illustrator 2020 (Adobe Systems Inc., USA).

## Results

### Phylogenetic analysis

**Figure 1.**
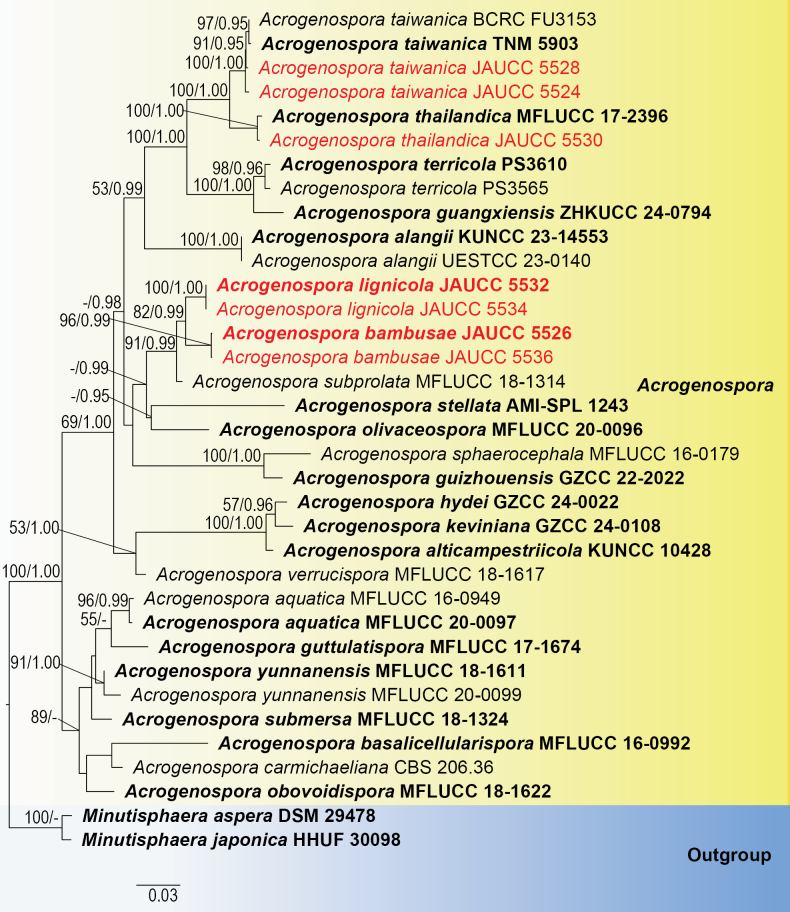
Phylogenetic tree reconstructed using RAxML based on concatenated sequences of the ITS, LSU, SSU, *RPB2*, and *TEF1-α* genetic markers. Nodes with bootstrap support values ≥50% for maximum likelihood analysis and Bayesian posterior probabilities ≥0.95 are indicated. The tree was rooted using *Minutisphaera aspera* (DSM 29478) and *Minutisphaera japonica* (HHUF 30098) as outgroups. Ex-type strains are highlighted in bold, and newly isolated strains from this study are marked in red.

A concatenated sequence matrix was constructed for phylogenetic analysis, comprising the following loci: ITS (615 bp), LSU (1391 bp), SSU (950 bp), *RPB2* (1026 bp), and *TEF1-α* (919 bp), resulting in a total alignment length of 4901 characters, including gaps. The topologies of the phylogenetic trees reconstructed using Maximum Likelihood (ML) and Bayesian Inference (BI) were congruent. The best-fit tree generated by RAxML, with bootstrap support (BS) and posterior probability (PP) values, is presented in Fig. 1. Two isolates of *Acrogenospora bambusae* (JAUCC 5526 and 5536) formed a monophyletic clade (ML = 96%, PP = 0.99), while two isolates of *Acrogenospora lignicola* (JAUCC 5532 and 5534) constituted another distinct clade (ML = 100%, PP = 1.00). Together, these two clades formed a supported larger clade (ML = 82%, PP = 0.99), which is phylogenetically sister to *Acrogenospora subprolata* (ML = 91%, PP = 0.99). Furthermore, phylogenetic analysis confirmed that the newly sequenced isolates of *A. taiwanica* (JAUCC 5524 and 5528) clustered strongly (ML = 100%, PP = 1.00) with the existing reference specimens of *A. taiwanica*: the ex-type strain TNM 5903 and the isolate BCRC FU3153. Similarly, the new isolate *A. thailandica*JAUCC 5530 formed a well-supported clade with the ex-type strain of *A. thailandica*MFLUCC 17-23976 (ML = 100%, PP = 1.00).

### Taxonomy

#### Acrogenospora bambusae

Taxon classificationFungiMinutisphaeralesAcrogenosporaceae

J.X. Nie & Z.J. Zhai sp. nov.

615EBDCE-C821-5495-A313-F24E7949F93D

857733

Fig. 2

##### Holotype.

HFJAU 10107.

##### Etymology.

The epithet ‘bambusae’ refers to the host on which the holotype was collected.

##### Description.

Saprobic on submerged decaying bamboo stem in freshwater. ***Sexual morph***: Undetermined. ***Asexual morph***: Hyphomycetous. ***Colonies*** on natural substrate, effuse, hairy, black, glistening. ***Mycelium*** mostly immersed in the substratum, consisting of hyaline to brown, branched, smooth, septate, 2–4.5 μm wide hyphae. ***Conidiophores*** 254–533 × 5.5–13 μm (x̄ = 345.6 × 8.5, *n* = 30), macronematous, mononematous, solitary or in groups, erect, straight or slightly curved, occasionally branched, light brown to black, apically tapering, septate, smooth. ***Conidiogenous cells*** holoblastic, monoblastic, integrated, medium brown, terete, sometimes curved at the point of proliferation, percurrent proliferation. ***Conidia*** 33–52 × 24–41 µm (x̄ = 39.5 × 29, *n* = 50), acrogenous or acropleurogenous, solitary, dry, mostly oval to broadly ellipsoidal, base truncate, aseptate, hyaline when young, orange-brown at maturity, thick-walled, smooth, occasionally with a large guttule.

**Figure 2.**
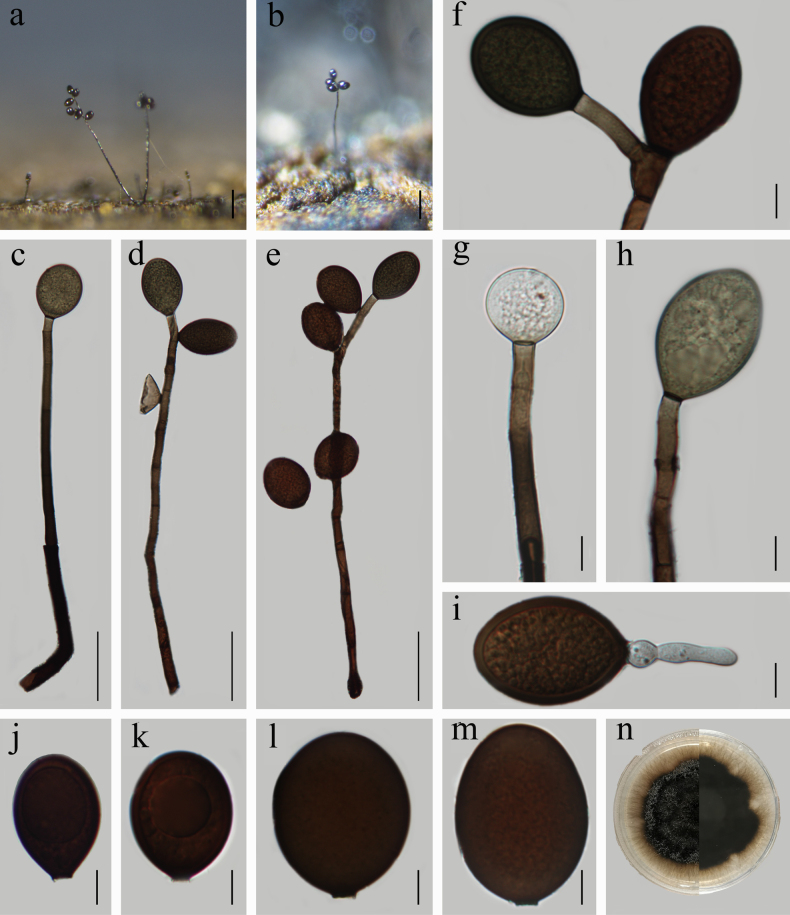
*Acrogenospora bambusae* (HFJAU 10107, holotype). **a, b**. Colonies on bamboo; **c–h**. Conidiophores with conidia; **j–m**. Conidia; **i**. Germinated conidium; **n**. Culture on PDA from above (left) and reverse (right). Scale bars: 100 µm (**a, b**); 50 µm (**c–e**); 10 µm (**f–m**).

##### Cultural characteristics.

Conidia germinating on PDA within 24 hours and germ tubes produced from the conidial base. Colonies on PDA growing slowly, reaching 32 mm diam in 24 days at 25 °C in natural light, rounded, aerial hyphae black, reverse black, entire.

##### Material examined.

China • Jiangxi Province, Jiujiang City, Yongxiu County, Yunjushan Mountain, 29.23°N, 115.59°E, at an altitude of 672.5 m, on decaying bamboo stem submerged in a freshwater stream, 28 April 2020, Z. J. Zhai, YJS-65 (HFJAU 10107, holotype), ex-type living culture JAUCC 5526; China • Guizhou Province, Tongren City, Jiangkou County, Fanjing Mountain, 27.91°N, 108.70°E, at an altitude of 597 m, on decaying woody substrate submerged in a freshwater stream, 10 October 2022, Z. J. Zhai, FJS-11 (HFJAU 10117, paratype), ex-paratype living culture JAUCC 5536.

##### Notes.

In the multi-gene phylogenetic analysis, *Acrogenospora bambusae* (JAUCC 5526, JAUCC 5536) and *A. lignicola* cluster together with well-supported (82% ML, 0.99 PP; Fig. 1) statistical values as a sister lineage to *A. subprolata* (MFLUCC 18-1314). Pairwise base pair comparisons in the currently available LSU and SSU gene regions revealed 12 (0.92%, excluding gaps) and one (0.13%, excluding gaps) nucleotide differences between *A. bambusae* and *A. subprolata* (MFLUCC 18-1314), respectively. Comparisons using the combined multi-gene matrix (ITS–LSU–SSU–*RPB2*–*TEF1-α*) showed that *A. bambusae* exhibits 130 nucleotide differences (2.79%, including two gaps) relative to *A. lignicola*. Morphologically, *A. bambusae* is most similar to *A. ovalia*, *A. stellata*, and *A. subprolata* in having acrogenous or acropleurogenous, solitary, mostly oval to broadly ellipsoidal, and thick-walled conidia. However, our new collection differs from *A. ovalia* and *A. stellata* by its conidial size (33–52 × 24–41 µm vs. 24–33 × 18–22 and 25–30 × 15–20 μm), respectively (Goh et al. 1998; Tan et al. 2022), and differs from *A. subprolata* by its longer (254–533 µm vs. 150–300 µm) conidiophores (Goh et al. 1998). Additionally, *A. bambusae* differs from *A. lignicola* in having different conidial shapes (mostly oval to broadly ellipsoidal vs. mostly broadly obovoid) and colors (hyaline when young, orange-brown at maturity vs. dark brown to black). More importantly, *A. bambusae* is distinct from *A. lignicola*, *A. ovalia*, *A. stellata*, and *A. subprolata* in our phylogenetic tree. Therefore, *A. bambusae* is introduced as a new taxon within *Acrogenospora* based on morphology and phylogeny. A comprehensive morphological comparison with related *Acrogenospora* species is provided in Table 2.

#### Acrogenospora lignicola

Taxon classificationFungiMinutisphaeralesAcrogenosporaceae

J.X. Nie & Z.J. Zhai sp. nov.

8553AC8F-CC9D-5F1B-9124-1927E970ED13

857734

Fig. 3

##### Etymology.

Referring to this taxon dwelling on woody substrate.

##### Description.

Saprobic on rotting wood in freshwater. ***Sexual morph***: Undetermined. ***Asexual morph***: Hyphomycetous. ***Colonies*** on natural substratum, effuse, hairy, glossy. ***Mycelium*** submerged in substrate for the most part, composed of septate, brown, branched, smooth, 2–4 µm wide hyphae. ***Conidiophores*** 149.5–352 × 4.5–7.5 µm (x̄ = 241 × 6, *n* = 30), macronematous, mononematous, unbranched, mostly flexuous, brown to dark brown, smooth, uniformly colored, septate, slightly broader at the base, tapering toward the apex. ***Conidiogenous cells***, holoblastic, monoblastic, integrated, initially terminal, later becoming intercalary, cylindrical, smooth, pale brown, proliferating percurrently. ***Conidia*** 26–45 × 22–36 µm (x̄ = 35.5 × 29, *n* = 50), acrogenous or acropleurogenous, solitary, dry, mostly broadly obovoid, dark brown to black, sometimes with several small guttules, thick-walled, smooth.

**Figure 3.**
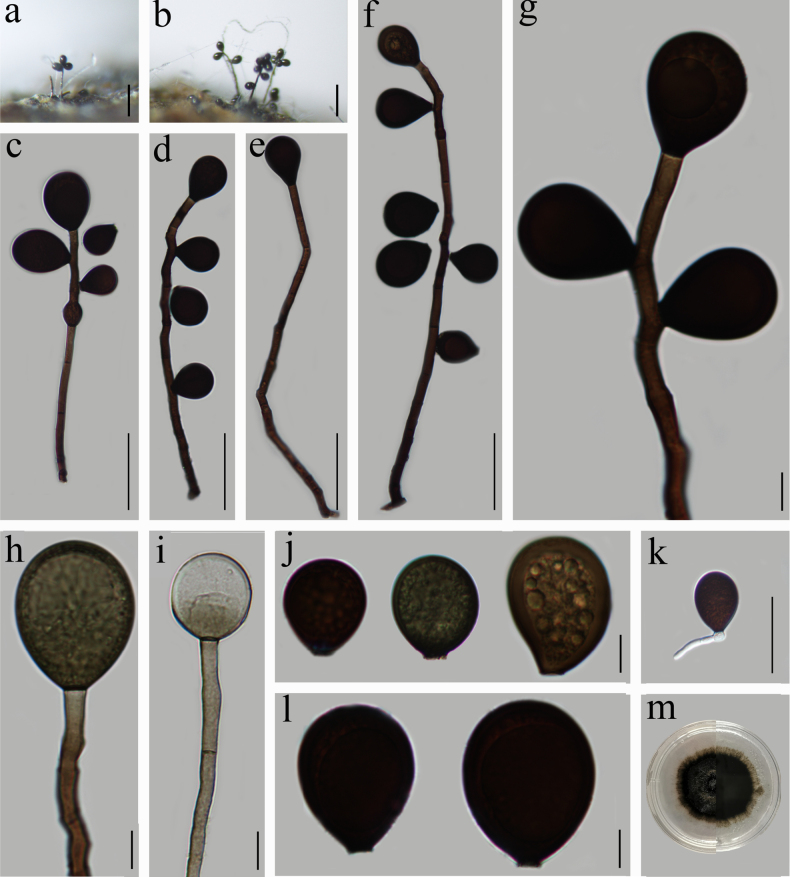
*Acrogenospora lignicola* (HFJAU 10113, holotype). **a, b**. Colonies on wood; **c–i**. Conidiophores with conidia; **j, l**. Conidia; **k**. Germinated conidium; **m**. Culture on PDA from above (left) and reverse (right). Scale bars: 100 µm (**a, b**); 50 µm (**c–f, k**); 10 µm (**g–j, l**).

##### Cultural characteristics.

Conidia germinating on PDA within 24 hours and germ tubes produced from the conidial base. The colonies on PDA grown slowly, attaining a diameter of 24.5 mm after 24 days at 25 °C under natural light conditions. The spore was round, hairy, circularly dispersed, regular; the adaxial surface was outwardly expanded, slightly projected, with dense aerial mycelium, black, reverse surface black, margin diffuse.

##### Material examined.

China • Jiangxi Province, Yichun City, Fengxin County, Dagang Forest Farm, 28.627°N, 114.943°E, at an altitude of 362 m, on decaying wood submerged in a freshwater stream, 28 April 2020, Z. J. Zhai, DGS-15 (HFJAU 10113, holotype), ex-type living culture JAUCC 5532; 6 November 2022, Z. J. Zhai, DGS-36 (HFJAU 10118), ex-paratype living culture JAUCC 5534.

##### Notes.

In the phylogenetic analysis, *Acrogenospora lignicola* (JAUCC 5532, JAUCC 5534) distinctly clusters with *A. bambusae* (JAUCC 5526, JAUCC 5536) with moderate statistical support (82% ML, 0.99 PP; Fig. 1). However, as mentioned above, *A. lignicola* can be easily distinguished from *A. bambusae* by its conidial shape and conidial color (Table 2). Morphologically, *Acrogenospora lignicola* is most similar to *A. basalicellularispora* and *A. obovoidispora* in having oval to broadly obovoid conidia (Bao et al. 2020). However, *A. lignicola* can be distinguished from them by its thinner conidiophores (4.5–7.5 µm vs. 8–12 µm and 7.5–10 µm). Furthermore, *A. lignicola* differs from *A. basalicellularispora* by its dark brown to black conidia that lack a basal cell, whereas *A. basalicellularispora* has pale orange-brown to olivaceous brown conidia with a small, hyaline, subcylindrical to subglobose basal cell (Bao et al. 2020). According to phylogenetic and morphological evidence, we introduce *A. lignicola* as a new species in *Acrogenospora*.

#### Acrogenospora taiwanica

Taxon classificationFungiMinutisphaeralesAcrogenosporaceae

Roland Kirschner, Taiwania 70(1): 125–131 (2025)

3D23F00C-2FD3-57B0-AB58-23C2527924FD

Fig. 4

##### Description.

Saprobic on rotting wood in freshwater. ***Sexual morph***: Undetermined. ***Asexual morph***: Hyphomycetous. ***Colonies*** on natural substrate, hairy, sparse, black. ***Mycelium*** mostly submerged, 1.5–3 µm wide, composed of hyaline to brown septate hyphae. ***Conidiophores*** 92.5–417 × 5–7.5 µm (x̄ = 235.5 × 6, *n* = 30), solitary, straight or slightly curved, septate, unbranched, cylindrical, dark brown, paler to the apex, smooth. ***Conidiogenous cells*** monoblastic, integrated, initially terminal, later becoming intercalary, cylindrical, smooth, brown. ***Conidia*** 21–32.5 µm (x̄ = 27, *n* = 50) diam., solitary, terminal or lateral, globose or subglobose, hyaline when young, dark brown when mature, smooth, thick-walled, aseptate, sometimes with a large guttule, base truncate.

**Figure 4.**
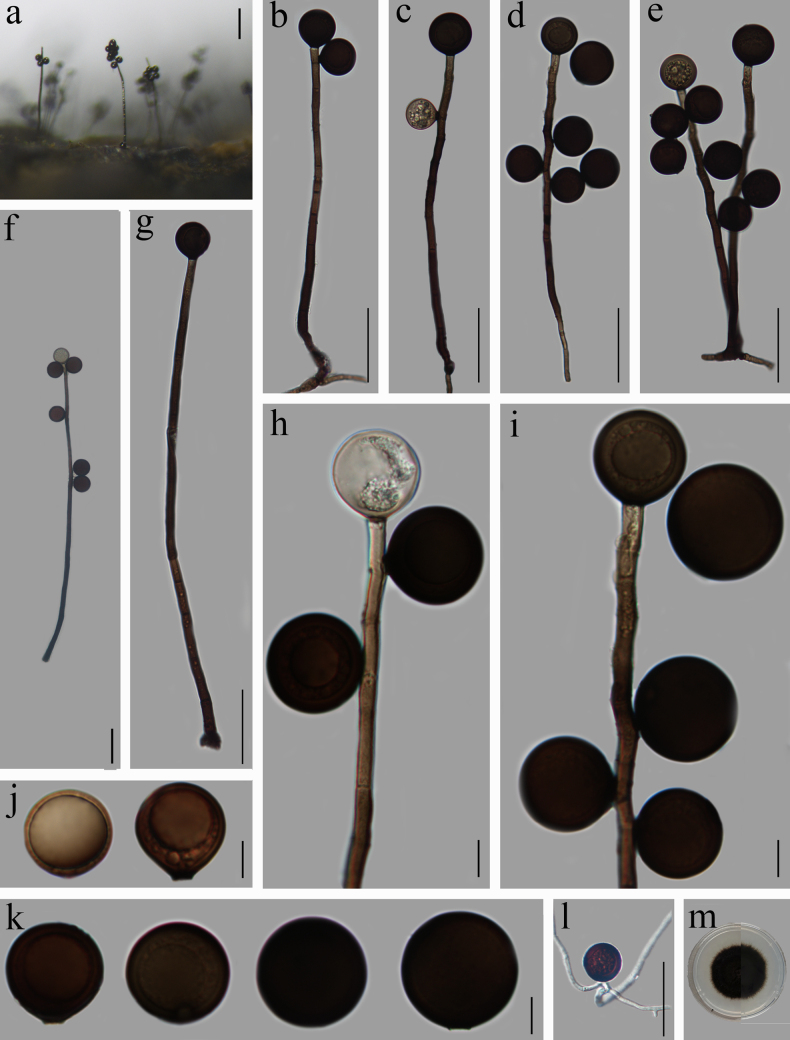
*Acrogenospora taiwanica* (HFJAU 10105). **a**. Colonies on wood; **b–i**. Conidiophores with conidia; **j, k**. Conidia; **l**. Germinated conidium; **m**. Culture on PDA from above (left) and reverse (right). Scale bars: 100 µm (**a**); 50 µm (**b–g, l**); 10 µm (**h–k**).

##### Cultural characteristics.

Conidia germinating on PDA within 24 hours and germ tubes produced from the conidial base. Colonies cultivated on PDA exhibited slow growth, attaining a diameter of 23 mm over 24 days at 25 °C under natural light. The spore exhibited a round and hairy morphology, with a dense aerial mycelium covering the surface. The margins of the regular mycelium appear slightly dispersed, while both the anterior and posterior surfaces are consistently rounded and exhibit a black coloration.

##### Material examined.

China • Jiangxi Province, Jiujiang City, Yongxiu County, Yunjushan Mountain, 29.23°N, 115.59°E, at an altitude of 672.5 m, on decaying wood submerged in a freshwater stream, 28 April 2020, Z. J. Zhai, YJS-30 (HFJAU 10105), living culture JAUCC 5524; 4 May 2021, Z. J. Zhai, YJS66 (HFJAU 10229), living culture JAUCC 5528.

##### Notes.

Phylogenetic analysis revealed that our new isolates (JAUCC 5524 and JAUCC 5528) clustered together (100% ML, 1.00 PP; Fig. 1) with *Acrogenospora taiwanica* (BCRC FU3153 and TNM 5903). At the ITS locus, the newly isolated strains JAUCC 5524 and JAUCC 5528 showed four and six nucleotide differences (excluding gaps), respectively, compared to the holotype of *A. taiwanica*TNM 5903, while exhibiting only two and four (excluding gaps) differences relative to *A. taiwanica* BCRC FU3153 (Kirschner 2025). Furthermore, our two strains showed no variation in their LSU sequences and were identical to *A. taiwanica* BCRC FU3153. Morphologically, our new collections resemble the holotype of *A. taiwanica*, except for the slightly larger (21–32.5 μm vs. 21.5–25 μm) conidia (Kirschner 2025). The difference in conidial size might be due to factors such as habitat and incubation time (Yang et al. 2018; Zhang et al. 2022). *Acrogenospora taiwanica* was previously found on dead terrestrial wood from Taiwan Province, China, and our new collections were collected from submerged, decaying wood in Jiangxi Province, which is a new discovery in freshwater habitats in mainland China.

#### Acrogenospora thailandica

Taxon classificationFungiMinutisphaeralesAcrogenosporaceae

J. Yang & K.D. Hyde, Fungal diversity 96: 78 (2019)

8C94CB37-D944-56BB-A499-95ADC54BBDB5

Fig. 5

##### Description.

Saprobic on rotting wood in freshwater. ***Sexual morph***: Undetermined. ***Asexual morph***: Hyphomycetous. ***Colonies*** sparse, scattered, hairy, black, glossy. ***Mycelium*** mostly submerged, composed of black, branched, smooth, 2–4 µm wide hyphae. ***Conidiophores*** 204.5–506 × 5–9.5 µm (x̄ = 399.5 × 7, *n* = 30) macronematous, mononematous, solitary, erect or curved, pale to dark brown, paler towards the apex, septate, smooth. ***Conidiogenous cells*** holoblastic, monoblastic, integrated, pale brown, cylindrical, integrated. ***Conidia*** 21–34.5 µm (x̄ = 25.5, *n* = 50) diam., thick-walled, smooth, spherical or subspherical, Olive-green to dark brown, sometimes with a large guttule, truncate at the base.

**Figure 5.**
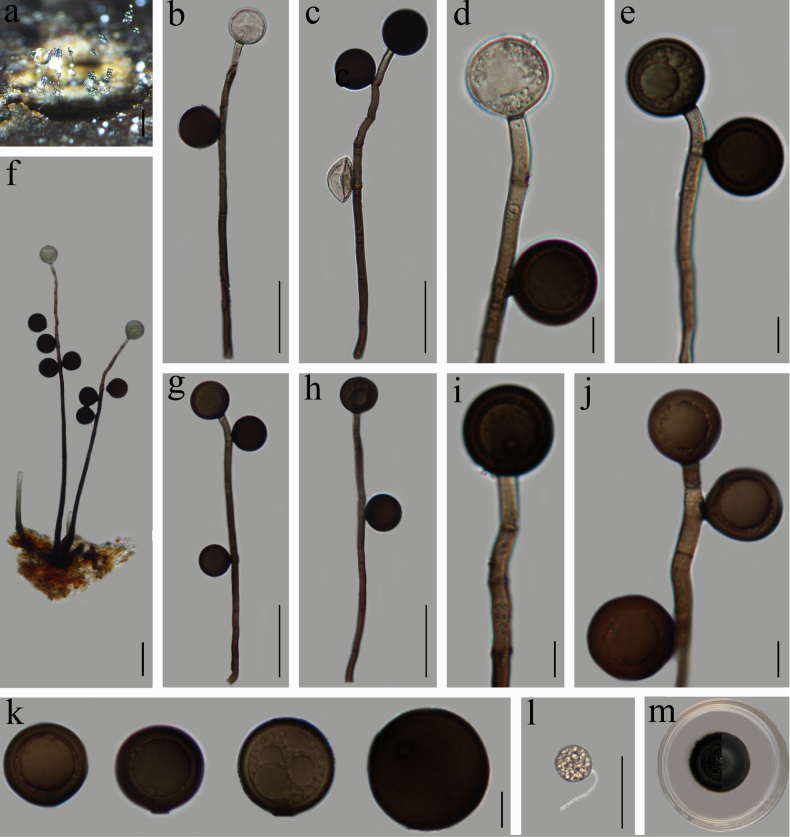
*Acrogenospora thailandica* (HFJAU 10111). **a**. Colonies on wood; **b–j**. Conidiophores with conidia; **k**. Conidia; **l**. Germinated conidium; **m**. Culture on PDA from above (left) and reverse (right). Scale bars: 200 µm (**a**); 50 µm (**b, c, f–h, l**); 10 µm (**d, e, i, j, k**).

##### Cultural characteristics.

Conidia germinating on PDA within 24 h and germ tubes produced from the conidial base. Colonies on PDA growing slowly, reaching 23 mm diam in 24 days at 25 °C in natural light, round, hairy, surface with dark grey-green aerial mycelium, margin entire.

##### Material examined.

China • Jiangxi Province, Yichun City, Fengxin County, Dagang Forest Farm, 28.627°N, 114.943°E, at an altitude of 362 m, on decaying wood submerged in a freshwater stream, 5 May 2020, Z. J. Zhai, DGS-2 (HFJAU 10111), living culture JAUCC 5530.

##### Notes.

Phylogenetic analysis of the combined ITS, LSU, SSU, *RPB2*, and *TEF1-α* sequences showed that our new strain JAUCC 5530 clustered together with the ex-type strain of *A. thailandica* (MFLUCC 17-2396) with high statistical support (100% ML, 1.00 PP; Fig. 1). Morphologically, JAUCC 5530 shared similar conidial size with MFLUCC 17-2396 but possessed shorter (204.5–506 μm vs. 850–950 μm) conidiophores (Hyde et al. 2019). However, there were only 0.21% (1/472 bp, excluding gaps) nucleotide differences in ITS sequences, 0.72% (6/828 bp, excluding gaps) in LSU sequences, no differences in SSU sequences, and 0.58% (6/1026 bp, excluding gaps) in *RPB2* sequences. Therefore, we identified our isolate as *A. thailandica*. The holotype of *A. thailandica* was originally described from submerged decaying wood in a freshwater habitat in Thailand. In the present study, this species is newly reported from submerged decaying wood in freshwater habitats in Jiangxi Province, China, representing its first record from the country.

## Discussion

Since the early 1970s, mycologists have investigated the diversity of the genus *Acrogenospora* (Ellis 1971, 1972). Early studies relied heavily on morphological characteristics, resulting in an inaccurate taxonomic status for many species, such as *Acrogenospora altissima* (originally described as *Monotospora altissima*) and *A. carmichaeliana* (initially misidentified as *Hysterium carmichaelianum*) (Hughes et al. 1978; Goh et al. 1998; Rossman et al. 2015). Over the past decade, the genus has been studied more intensively using combined morphological and multi-gene phylogenetic analyses based on LSU, SSU, *RPB2*, and *TEF1-α* sequence data (Harrington et al. 2022; Cao et al. 2025; Xu et al. 2025). However, these studies often omitted ITS sequences, which reveal significant nucleotide differences between species and are crucial for species-level identification, despite being unavailable for many taxa. This study integrates morphological observations with multi-locus phylogenetic analyses of ITS, LSU, SSU, *RPB2*, and *TEF1-α* gene regions. The results identified two novel species and two new records of *Acrogenospora* in mainland China. For instance, *Acrogenospora bambusae* and *A. lignicola* are distinguished as distinct species, forming well-supported, separate clades in the phylogeny (Fig. 1) and exhibiting clear diagnostic morphological differences.

Members of the genus *Acrogenospora* have a global distribution, with records from Asia, Europe, America, and Oceania (including China, Thailand, the United Kingdom, Portugal, Canada, Panama, New Zealand, and Australia; see Table 2). Currently, over 50% of known *Acrogenospora* species have been discovered in freshwater or terrestrial environments in China. This pattern is likely associated with the country’s vast territory and diverse landscapes and climates, which harbor exceptionally high fungal diversity (Liu et al. 2025). The taxa include *A. alangii*, *A. alticampestriicola*, *A. aquatica*, *A. basalicellularispora*, *A. ellipsoidea*, *A. guizhouensis*, *A. guttulatispora*, *A. hainanensis*, *A. hydei*, *A. keviniana*, *A. obovoidispora*, *A. olivaceospora*, *A. ovalia*, *A. sphaerocephala*, *A. submersa*, *A. subprolata*, *A. taiwanica*, *A. verrucispora*, and *A. yunnanensis* (Goh et al. 1998; Ho et al. 2001; Zhu et al. 2005; Hu et al. 2010; Ma et al. 2012; Bao et al. 2020; Hyde et al. 2023; Li et al. 2024; Cao et al. 2025; Kirschner 2025; Xiao et al. 2025; Xu et al. 2025). Within China, most species have been reported from the southwestern provinces of Yunnan and Guizhou, with fewer records from Hainan, Hong Kong, Guangxi, Taiwan, and Xizang. This study describes a new species from Guizhou Province and reports three additional isolates from Jiangxi Province, representing a new distribution record for the genus. These findings indicate that the diversity and distribution of *Acrogenospora* in China are likely far broader than currently recognized. However, to date, only 31 species (including two described in this study) have been formally documented worldwide. This significant gap between observed distribution and described diversity is likely due to limited collection efforts and field investigations, suggesting that many more *Acrogenospora* species await discovery.

## Supplementary Material

XML Treatment for Acrogenospora bambusae

XML Treatment for Acrogenospora lignicola

XML Treatment for Acrogenospora taiwanica

XML Treatment for Acrogenospora thailandica

## Additional information

### Use of AI

No use of AI was reported.

### Author ORCIDs

Mei-Xia Wang https://orcid.org/0000-0002-0068-2449

Jia-Xin Nie https://orcid.org/0009-0005-7101-5431

Ye-Tao Wang https://orcid.org/0009-0006-9697-293X

Dian-Ming Hu https://orcid.org/0000-0002-4750-2871

Zhi-Jun Zhai https://orcid.org/0009-0008-7562-9707

### Data availability

All of the data that support the findings of this study are available in the main text or Supplementary Information.

## Supplementary materials

Supplementary material 1 its-lsu-ssu-rpb2-tef1 Data type fas https://binary.pensoft.net/file/1570154This dataset is made available under the Open Database License (http://opendatacommons.org/licenses/odbl/1.0/). The Open Database License (ODbL) is a license agreement intended to allow users to freely share, modify, and use this Dataset while maintaining this same freedom for others, provided that the original source and author(s) are credited. Mei-Xia Wang, Jia-Xin Nie, Ye-Tao Wang, Min Xiong, Jing Wang, Hai-Yan Song, Dian-Ming Hu, Zhi-Jun Zhai
